# Cancer symptom and risk factor awareness in Malaysia: findings from a nationwide cross-sectional study

**DOI:** 10.1186/s12889-020-08581-0

**Published:** 2020-04-06

**Authors:** Désirée Schliemann, Roshidi Ismail, Michael Donnelly, Christopher R. Cardwell, Tin Tin Su

**Affiliations:** 1 Centre for Public Health and UKCRC Centre of Excellence for Public Health, Queen’s University Belfast, Belfast, UK; 2 South East Asia Community Observatory (SEACO), Jeffrey Cheah School of Medicine and Health Sciences, Monash University Malaysia, Bandar Sunway, Malaysia; 3 Centre for Population Health (CePH), Department of Social and Preventive Medicine, Faculty of Medicine, University of Malaya, Kuala Lumpur, Malaysia

**Keywords:** Cancer awareness, Signs, Symptoms, Risk factors, Malaysia, ABC measurement tool

## Abstract

**Background:**

Cancer incidence in Malaysia is expected to double by 2040. Understanding cancer awareness is important in order to tailor preventative efforts and reduce the cancer burden. The objective of this research was to assess nationwide awareness about the signs and symptoms as well as risk factors for various cancers in Malaysia and identify socio-demographic factors associated with awareness.

**Methods:**

This cross-sectional study was conducted from March–November 2014 in the form of a telephone survey. Participants aged 40 years and above were randomly selected across Malaysia and interviewed using the validated Awareness Beliefs about Cancer (ABC) measurement tool. Linear regression was conducted to test the association between symptom and risk factor recognition and socio-demographic variables.

**Results:**

A sample of 1895 participants completed the survey. On average, participants recognised 5.8 (SD 3.2) out of 11 symptoms and 7.5 (SD 2.7) out of 12 risk factors. The most commonly recognised symptom was ‘lump or swelling’ (74.5%) and the most commonly recognised risk factor was ‘smoking’ (88.7%). Factors associated with prompted awareness were age, ethnicity, education and smoking status.

**Conclusion:**

Recognition of symptom and risk factors for most cancers was relatively low across Malaysia compared to previous studies in high-income countries and to studies conducted in Malaysia. There is a need to conduct regular public health campaigns and interventions designed to improve cancer awareness and knowledge as a first step towards increasing the early detection of cancer.

## Background

Increasingly, Malaysians are adopting behaviours associated with western lifestyles and linked to increased cancer risk. South East Asia, including Malaysia, has one of the highest intake of saturated fatty acids globally [1]. Sugar, sweetened condensed milk and local sweets are consumed commonly in Malaysia [2] and over 50% of Malaysians are overweight or obese [3]. Furthermore, according to the World Health Organisation, over 40% of Malaysian males were smokers in 2016 [4]. These types of behaviours have been identified as contributing risk factors to the rapid increase in cancer burden in Malaysia. According to GLOBOCAN 2018, cancer incidence in Malaysia is expected to double by 2040 (from 43,837 to 84,158 cases) [5]. In countries such as the UK, it is estimated that 4 in 10 cancer cases could be prevented by addressing these modifiable lifestyle factors [6]. Therefore, raising awareness about risk factors is a first step towards cancer prevention followed by interventions that tackle risk behaviours.

The few cancer awareness studies that have been conducted in Malaysia to date generally focused on one state and/or a particular type of cancer; and paid insufficient attention to symptom awareness [7, 8]. Thus, an understanding about nationwide levels of awareness regarding common risk factors and symptoms for cancer is lacking, including the nature and extent of variation. Poor awareness and knowledge about the signs and symptoms of common cancers is likely to contribute to a delay in help-seeking [9], diagnosis and treatment and, in turn, lead to poor survival outcomes [10]. There is a need to investigate and understand awareness levels and knowledge gaps in order to tailor health promotion interventions that will reduce cancer incidence and improve early detection and outcomes [11].

The *Awareness and Beliefs about Cancer* (ABC) measurement tool was developed and tested in a six-country international benchmarking study [12]. Recently, the ABC tool was culturally adapted and validated for use in Malaysia (*manuscript in preparation*). The use of a gold standard assessment tool such as the ABC aids global health efforts by providing cross-country, comparative analytical data with which to ‘benchmark’ cancer awareness levels and inform public health cancer reduction strategies and interventions. Therefore, the main objective of this research was to provide a nationwide empirical assessment of awareness about cancer warning signs and risk factors in Malaysia using an internationally validated measurement tool. In addition, the research investigated differences in awareness levels between population groups in order to aid the development and tailoring of future cancer education efforts that take account of cultural differences and variation according to socio-demographic factors.

## Methods

This study was a cross-sectional, nationwide telephone-conducted interview survey of randomly selected residents in Malaysia.

### Study setting and population

Malaysia is an upper middle-income country located in South East Asia and is made up of 13 states and three federal territories. The three main ethnic groups in Malaysia are Malays (69.1%), Chinese (23.0%) and Indians (6.9%) [13]. Participants were selected randomly to represent people living across all states of Malaysia who met the following inclusion criteria: I) able to understand and speak Bahasa Melayu, Mandarin, Tamil or English and II) had a mobile phone number that was randomly selected as described below and III) were aged 40 years or above. This age group was chosen as cancer incidence is higher in people aged 40 years and above in Malaysia.

### Sampling

Mobile phone numbers were purchased by the researchers in August 2013 from Sample Solutions, a company providing telephone numbers for research purposes. In total, 150,000 phone numbers were generated at random taking into account operator prefixes. The mobile phone numbers were then screened for an activated SIM card. Inactive mobile phone numbers were removed from the sample and around 55,000 mobile phone numbers were retained. Trained research assistants called 8000 active phone numbers and 1895 participants met the inclusion criteria and agreed to participate.

### Measurement tool

The previously validated and culturally adapted ABC questionnaire tool was used to assess awareness about cancer-related symptoms and risk factors [12]. The tool assessed awareness and knowledge about eleven cancer-related symptoms and twelve risk factors for cancer. Knowledge was assessed through unprompted questions, i.e. participants were asked *‘There are many warning signs and symptoms of cancer. Please name as many as you can think of.’*, whereas awareness of symptoms was tested through prompted questions, i.e. participants were told, *‘I’m now going to list some symptoms that may or may not be warning signs for cancer. For each one, can you tell me whether you think that it could be a warning sign for cancer’*. Similarly, prompted awareness about risk factors was assessed by asking ‘*I am now going to read out a list of things which may or may not increase your chances of getting cancer in general. For each one can you tell me how much you agree or disagree that it may increase your chances of getting cancer?’* with the possible answers ranging from strongly agree to strongly disagree on a 5-point Likert scale. Unprompted knowledge about risk factors was not assessed as risk factor awareness was optional in the original ABC and the study team wanted to avoid overburdening participants. Socio-demographic and health-related information (i.e. gender, age, ethnicity, education, marital status, household ownership of motorised transport, smoking, access to doctors, experience of cancer, self-reported health) was assessed as part of the ABC survey.

### Interviews

Telephone interviews were conducted from March until November 2014 by trained research staff with different language skills (English/ Bahasa Melayu/ Mandarin/ Tamil) who called participants between 11 am and 7 pm from Monday to Friday and between 12 noon and 6 pm on Saturday and Sunday. Respondents who agreed to participate were asked for their preferred language for the conduct of the interview. Appointments at a later time or day were arranged whenever respondents were not able to participate on first contact or if the interviewer in the preferred language was not available. The telephone stations were set up using computer-assisted telephone interview (CATI) software (www.surveysystem.com/interviewing-cati.htm). The ABC Malaysia questionnaire was set up in the CATI software together with an introductory text and interview scripts for the research staff to follow in all four languages. For prompted questions, answer options were provided and interviewers clicked on the appropriate answer given by a respondent or typed verbatim the answers given to unprompted questions. The CATI software was set up so that the order in which symptom items appeared was random, e.g. ‘an unexplained lump or swelling’ might appear first on the list in one interview session, and then third on the list to a different respondent to minimize question order bias. This was also the case for risk factors.

### Sample size

It was estimated that a sample size of approximately 2000 would produce relatively precise national and sub-group estimates. For example, a study of colorectal cancer awareness in a multi-ethnic rural population in Perak, Malaysia, reported that 33.1% of the study population had knowledge about ‘unexplained weight loss’ and 27.7% about ‘unexplained tiredness’ as a warning sign for cancer [7]. Sample size calculations indicated that a sample of around 2000 participants would allow the proportion with knowledge about ‘unexplained weight loss’ and ‘unexplained tiredness’ to be estimated within plus or minus approximately 2% with 95% probability; and detect (with 90% power) a Malay vs non-Malay difference of 7% in the proportion of people with unexplained weight loss.

### Statistical analysis

Data entered in CATI was automatically compiled in an Excel spreadsheet, which was transferred into Stata SE 13.0 for statistical analysis. Symptom and risk factor scores were aggregated for constructs with more than one question. For example, there were eleven items related to symptoms awareness and twelve related to risk factor awareness. New variables called ‘number of recognised signs’ and ‘number of recognised risk factors’ were created by adding the number of signs/ risk factors. Descriptive statistics including socio-demographics and cancer symptoms and risk factor knowledge are presented as means and standard deviation (SD) for continuous variables and as frequencies and percentage (%) for categorical variables. Linear regression was conducted to assess the association between symptom recognition score and socio-demographic variables. The final model contained gender, age groups (in categories shown in Table 1), ethnicity, education level, marital status, household ownership of motorised vehicle, smoking status, self-reported health, access to doctors and experience of cancer.

**Table 1:** Socio-demographic and health-related characteristics of the study population (*n* = 1895)

	*n*	%
**Sex**		
Male	1082	57.1
Female	813	42.9
**Age groups**		
40–49	1279	67.5
50–59	427	22.5
60+	189	10.0
**Ethnicity**		
Malay	925	48.8
Chinese	754	39.8
Indian	162	8.5
Others	54	2.8
**Highest Level of Education**		
No formal education	28	1.5
Primary education	162	8.6
Secondary education	1052	55.5
Tertiary education	653	34.5
**Marital Status**		
Married	1663	87.8
Not married	231	12.2
**Household ownership of motorised transport**		
No	89	4.7
Yes, one	1043	55.0
Yes, more than one	763	40.3
**Smoking**		
Current smoker	355	18.7
Ex-smoker	115	6.1
Never smoked	1425	75.2
**Access to doctors**		
Easy	1814	95.8
Difficult	80	4.2
**Experience of cancer** (self, family or friend)		
Yes	381	20.1
No	1511	79.9
**Self-reported health**		
Very good	110	5.8
Good	1262	66.6
Fair	481	25.4
Poor	40	2.1
Very poor	2	0.1

Missing information: Marital status (*n* = 1), Access to doctors (*n* = 1), Experience of cancer (*n* = 3), Self-reported health (*n* = 1)

## Results

### Socio-demographics

In total, 1895 participants were recruited (Table 1) from all 13 states in Malaysia. About half of the study population was male (57.1%), and the majority of the study population was aged between 40 to 49 years (67.5%), married (87.8%) and completed secondary or tertiary education (55.5 and 34.5%, respectively) (Table 1). The sample included all three ethnicities with a lower proportion of Malays and a higher proportion of Chinese compared to the national ethnic distribution (Malays 48.8%, Chinese 39.8% and Indians 8.5%). The majority of participants owned motorised transport (55% owned one vehicle; 40.3% owned more than one vehicle) and reported that they had easy access to health care (95.8%). Self-reported health was rated as ‘good’ by 66.6% of participants. Approximately one fifth of participants reported that they had cancer or that their close family and friends had cancer. Only 18.7% of participants reported that they were current smokers.

### Signs and symptoms awareness

There were marked differences between the number of cancer symptoms that were recalled (from unprompted questions) and symptoms that were recognised (from prompted questions) (Table 2). The number of symptoms that were recalled (unprompted awareness) was on average less than one symptom (Mean 0.2; SD 0.5) whereas average prompted awareness was 5.8 (SD 3.2) out of 11 symptoms (data not shown). ‘Lumps’ was the most commonly recalled symptom (unprompted awareness) (3.5%) followed by ‘unexplained tiredness’ (3.3%) and ‘persistent unexplained pain’ (3.1%). None of the participants recalled ‘difficulty swallowing’, ‘changing appearance of a mole’ or ‘unexplained night sweats’ as possible cancer warning signs. When prompted, recognition of individual symptoms ranged from 26.4% (unexplained night sweats) to 74.5% with ‘unexplained lump’ being the most commonly recognized symptom (74.5%), followed by ‘unexplained bleeding’ (65.5%), ‘persistent unexplained pain’ (63.9%) and ‘unexplained weight loss’ (57.5%).

**Table 2:** Knowledge about each cancer sign/ symptom and risk factor (prompted and unprompted awareness)

	Yes (n) prompted	%	Yes (n) unprompted	%
**Signs and symptoms**				
Unexplained lump or swelling	1412	74.5	67	3.5
Persistent unexplained pain	1210	63.9	59	3.1
Unexplained bleeding	1242	65.5	21	1.1
A persistent cough or hoarseness	1049	55.4	14	0.7
A change in bowel or bladder habits	953	50.3	9	0.5
A persistent difficulty swallowing	873	46.1	0	0
A change in appearance of a mole	847	44.7	0	0
A sore that does not heal	873	46.1	5	0.3
Unexplained night sweats	500	26.4	0	0
Unexplained weight loss	1090	57.5	50	2.6
Unexplained tiredness	890	47	62	3.3
**Risk factors**				
Smoking	1681	88.7	–	–
Exposure to another person’s smoke	1644	86.8	–	–
Drinking more than 1 unit of alcohol a day	1149	60.6	–	–
Eating less than 5 portions of fruits and vegetables a day	994	52.5	–	–
Eating red or processed meat once a day or more	1099	58	–	–
Being obese	1080	57	–	–
Getting sunburnt more than once as a child	765	40.4	–	–
Being over 70 years old	801	42.3	–	–
Having a close relative with cancer	1245	65.7	–	–
Infection with Human Papillomavirus	1001	52.8	–	–
Not doing much physical activity	1125	59.4	–	–
Exposure to radiation such as radioactive materials, x-rays or radon	1547	81.6	–	–

The univariate analyses showed that gender, age, ethnicity, education level, household ownership of motorised transport, smoking status, access to doctors and having personal experience of cancer were associated with the number of cancer warning signs and symptoms recognised (Table 3). The fully adjusted regression model (Table 3) showed that participants aged 60 years or older recognised − 0.63 (95% CI -1.14; − 0.12) fewer signs and symptoms compared to participants aged 40 to 49 years. Furthermore, participants from a Chinese ethnic background and ‘others’ (e.g. from indigenous population groups and neighbouring countries) were significantly less likely to recognise signs and symptoms compared to Malays by − 0.32 scores (95% CI -0.63; − 0.01) and − 2.01 scores (95% CI -2.09; − 0.26) respectively. Also, participants with primary, secondary and tertiary education were significantly more likely to recognise signs and symptoms compared to participants without formal education by 2.32 scores (95% CI 1.00; 3.64), 3.37 scores (95% CI 2.09; 4.65) and 3.72 scores (95% CI 2.41; 5.02), respectively, which equates to a greater mean symptoms awareness score of three to four symptoms. Participants who were not married recognised significantly fewer signs and symptoms compared to participants who were married by − 0.60 scores (95% CI -1.04; − 0.15). In addition, people who were ex-smokers and people who never smoked recognised significantly more signs and symptoms compared to current smokers by 0.98 scores (95% CI 0.31; 1.66) and 0.89 scores (95% CI 0.48; 1.31). Lastly, participants with no experience of cancer were significantly less likely to recognise signs and symptoms compared to participants who had experience with cancer by − 0.38 scores (95% CI -0.75; − 0.02).

**Table 3:** The relationship between the socio-demographic and health-related characteristics of survey respondents and their prompted cancer signs and symptoms awareness (linear regression)

				Multiple linear regression	
	Mean (SD)	Difference in mean (95% CI)	*p*	Adjusted difference in mean^a^ (95% CI)	*p*
**Gender**					
Male	5.6 (3.3)	*Reference*		*Reference*	0.297
Female	6.0 (3.2)	0.45 (0.15; 0.74)	0.003	0.18 (−0.15; 0.51)	
**Age**					
40–49 years	5.9 (3.2)	*Reference*		*Reference*	
50–59 years	5.9 (3.3)	0.04 (− 0.32; 0.39)	0.841	0.11 (− 0.25; 0.46)	0.556
60 + years	4.9 (3.5)	− 0.99 (−1.48; − 0.50)	< 0.001	−0.63 (−1.14; − 0.12)	0.016
**Ethnicity**					
Malay	5.9 (3.2)	*Reference*		*Reference*	
Chinese	5.7 (3.2)	−0.19 (− 0.50; 0.12)	0.238	− 0.32 (− 0.63; − 0.01)	0.044
Indian	5.9 (3.4)	− 0.02 (− 0.56; 0.52)	0.949	−0.14 (− 0.67; 0.39)	0.609
Others	4.3 (3.2)	− 1.60 (−2.48; − 0.71)	< 0.001	−2.01 (−2.09; − 0.26)	0.011
**Education level**					
No formal education	2.0 (2.1)	*Reference*		*Reference*	
Primary	4.6 (3.5)	2.55 (1.27; 3.83)	< 0.001	2.32 (1.00; 3.64)	< 0.001
Secondary	5.8 (3.3)	3.76 (2.57; 4.96)	< 0.001	3.37 (2.09; 4.65)	< 0.001
Tertiary	6.2 (2.9)	4.15 (2.94; 5.35)	< 0.001	3.72 (2.41; 5.02)	< 0.001
**Marital Status**					
Married	5.8 (3.3)	*Reference*		*Reference*	
Not married	5.4 (2.8)	−0.43 (−0.88; 0.01)	0.057	−0.60 (−1.04; − 0.15)	0.009
**Transport ownership**					
None	4.2 (3.5)	*Reference*		*Reference*	
Yes, one	5.8 (3.3)	1.60 (0.91; 2.30)	< 0.001	0.60 (− 0.12; 1.33)	0.102
Yes, more than one	5.9 (3.1)	1.68 (0.97; 2.39)	< 0.001	0.47 (− 0.28; 1.22)	0.219
**Smoking**					
Current smoker	4.9 (3.2)	*Reference*		*Reference*	
Ex-smoker	5.9 (3.2)	1.01 (0.33; 1.68)	0.004	0.98 (0.31; 1.66)	0.004
Never smoked	6.0 (3.2)	1.06 (0.68; 1.43)	< 0.001	0.89 (0.48; 1.31)	< 0.001
**Self-related health**					
Fair to very poor	5.9 (3.3)	*Reference*		*Reference*	
Good to very good	5.7 (3.2)	−0.13 (− 0.46; 0.20)	0.433	− 0.31 (− 0.63; 0.01)	0.060
Access to doctor					
Easy	5.8 (3.2)	*Reference*		*Reference*	
Difficult	5.0 (3.5)	−0.85 (−1.57; − 0.12)	0.022	− 0.54 (− 1.26; 0.18)	0.143
**Experience with cancer**					
Yes	6.1 (3.0)	*Reference*		*Reference*	
No	5.7 (3.2)	−0.42 (− 0.79; − 0.06)	0.022	− 0.38 (− 0.75; − 0.02)	0.041

^a^Model contains gender, age groups (in categories as shown in Table 1), ethnicity, education level, marital status, household ownership of motorised vehicle, smoking status, self-reported health, access to doctors and experience of cancer

### Risk factor awareness

On average, participants recognised (when prompted) 7.5 (SD 2.7) risk factors out of possible 12 risk factors (data not shown). Smoking was the most recognised risk factor for cancer (88.7%), followed by exposure to another person’s smoke (86.8%) and exposure to radiation such as radioactive materials, x-rays or radon (81.6%) (Table 2). About half of participants recognised life-style related risk factors such as drinking more than one unit of alcohol (60.6%), eating less than five portions of fruits and vegetables (52.5%), eating red or processed meat once a day or more (58.0%), being obese (57.0%) and being physically inactive (59.4%). Less than half of the respondents recognised older age (42.3%) and experiencing sunburn more than once as a child as risk factors (40.4%).

The univariate analysis showed that age, ethnicity, education level, smoking status and location were significantly associated with knowledge about cancer-related risk factors (Table 4). After full adjustment (Table 4), participants who were aged between 50 and 59 years had a lower prompted mean risk factor awareness score compared to participants aged between 40 and 49 years by − 0.44 scores (95% CI -0.74; − 0.14). Furthermore, all ethnic groups recognized significantly fewer risk factors compared to Malays (i.e. Chinese − 1.36, 95% CI -1.62; − 1.10 scores, Indian − 0.79, 95% CI -1.24; − 0.34 scores and ‘others’ -0.79, 95% CI -1.53; − 0.06 scores). Education level was also associated with number of risk factors recognised, i.e. participants who had primary, secondary or tertiary education recognised significantly more risk factors compared to participants with no formal education by 2.44 scores (95% CI 1.34; 3.55), 2.69 scores (95% CI 1.61; 3.76) and 2.95 scores (95% CI 1.85; 4.05), respectively. Participants who never smoked had a significantly higher mean prompted awareness score compared to current smokers by 0.68 scores (95% CI 0.34; 1.03).

**Table 4:** The relationship between the socio-demographic and health-related characteristics of survey respondents and their prompted cancer risk factor awareness (linear regression)

				Multiple linear regression	
	Mean (SD)	Difference in mean (95% CI)	*p*	Adjusted differnce in mean^a^ (95% CI)	*p*
**Gender**					
Male	7.4 (2.7)	*Reference*		*Reference*	0.716
Female	7.5 (2.8)	0.16 (− 0.09; 0.41)	0.201	− 0.05 (− 0.33; 0.23)	
**Age**					
40–49 years	7.6 (2.6)	*Reference*		*Reference*	
50–59 years	7.1 (3.0)	− 0.57 (− 0.86, − 0.27)	< 0.001	−0.44 (− 0.74; − 0.14)	0.003
60 + years	7.1 (2.8)	−0.58 (− 1.00; − 0.17)	0.006	−0.27 (− 0.70; 0.16)	0.22
**Ethnicity**					
Malay	8.0 (2.5)	*Reference*		*Reference*	
Chinese	6.8 (3.0)	−1.23 (− 1.49; −0.97)	< 0.001	−1.36 (− 1.62; − 1.10)	< 0.001
Indian	7.4 (2.3)	− 0.60 (− 1.05; − 0.15)	0.008	−0.79 (− 1.24; − 0.34)	< 0.001
Others	7.0 (2.6)	−1.03 (− 1.76; − 0.29)	0.006	−0.79 (− 1.53; − 0.06)	0.034
**Education level**					
No formal education	4.9 (3.5)	*Reference*		*Reference*	
Primary	7.2 (3.0)	2.31 (1.22; 3.40)	< 0.001	2.44 (1.34; 3.55)	< 0.001
Secondary	7.4 (2.8)	2.6 (1.56; 3.60)	< 0.001	2.69 (1.61; 3.76)	< 0.001
Tertiary	7.7 (2.5)	2.81 (1.78; 3.84)	< 0.001	2.95 (1.85; 4.05)	< 0.001
**Marital Status**					
Married	7.5 (2.8)	*Reference*		*Reference*	
Not married	7.5 (2.6)	0.03 (−0.35; 0.41)	0.868	−0.20 (−0.58; 0.17)	0.291
**Transport ownership**					
None	7.3 (3.2)	*Reference*		*Reference*	
Yes, one	7.5 (2.7)	0.24 (−0.35; 0.83)	0.428	−0.28 (− 0.89; 0.33)	0.369
Yes, more than one	7.4 (2.7)	0.15 (−0.45; 0.75)	0.633	−0.56 (−1.19; 0.07)	0.079
**Smoking**					
Current smoker	7.0 (2.8)	*Reference*		*Reference*	
Ex-smoker	7.5 (3.1)	0.45 (−0.13; 1.02)	0.128	0.35 (−0.21; 0.92)	0.218
Never smoked	7.6 (2.7)	0.56 (0.24; 0.88)	0.001	0.68 (0.34; 1.03)	< 0.001
**Self-related health**					
Fair to very poor	7.3 (2.8)	*Reference*		*Reference*	
Good to very good	7.5 (2.7)	0.26 (−0.02; 0.53)	0.066	0.23 (− 0.04; 0.50)	0.095
**Access to doctor**					
Easy	7.5 (2.7)	*Reference*		*Reference*	
Difficult	7.3 (2.9)	−0.11 (−0.73; 0.50)	0.719	−0.16; (− 0.76; 0.45)	0.612
**Experience with cancer**					
Yes	7.3 (3.0)	*Reference*		*Reference*	
No	7.5 (2.7)	0.18 (−0.13; 0.49)	0.249	−0.02 (− 0.29; 0.33)	0.902

^a^Model contains gender, age groups (in categories as shown in Table 1), ethnicity, education level, marital status, household ownership of motorised vehicle, smoking status, self-reported health, access to doctors and experience of cancer

## Discussion

Understanding cancer awareness of the public aids the design of cancer health education. To the best of our knowledge, this was the first nationwide survey to measure the level of awareness about general cancer signs and symptoms and cancer-related risk factors among the general population in Malaysia and to assess its association with socio-demographic characteristics. A breast cancer-related symptom (i.e. unexplained lump or swelling) was the only symptom recognised by about 75% of the study population and between 35 and 75% of the population were not aware of the other common cancer signs and symptoms. A previous study in Malaysia has shown higher awareness about cancer signs and symptoms for different cancers, e.g. a survey conducted during world digestive day with 2408 participants indicated that 86.6% of participants recognised ‘blood in stool’ as a sign for colorectal cancer and 83.4% recognised weight loss [14]. Another study looking at breast cancer signs and symptoms reported that women generally had good knowledge about breast cancer signs and symptoms (60–78%), however, did not recognised that ‘a painless breast lump’ was a sign for breast cancer (32.2%) [15]. A recent study on lung cancer awareness (*n* = 385) also showed relatively high symptoms recognition compared to the findings demonstrated in the current study (e.g. 89% recognised ‘worsening or change in existing cough’ as a symptom for lung cancer) [16]. One explanation for the marked differences in awareness reported between the current research and other studies may be that most studies have utilised convenience sampling rather than random sampling. This may explain why Su et al. [7] who also utilised random sampling were one of the few studies that reported similarly low awareness about colorectal cancer signs and symptoms. It may not be surprising that females were on average more aware about cancer-related signs and symptoms as breast cancer is the most commonly promoted cancer for cancer awareness raising campaigns run by non-government organisations and industry in Malaysia [17].

Knowledge about cancer risk factors concerning lifestyle and old age was low, which poses concerns due to earlier discussed lifestyle-related behaviours in Malaysia and the aging population, i.e. 9% of Malaysians is aged 65 years or older [18]. The low risk factor awareness (i.e. awareness about high consumption of red and processed meat and low consumption of fruits and vegetables) for colorectal cancer, the most common cancer in males (14.6%) and second most common cancer in females (11.1%) is similar to what previous studies have reported [7]. Cervical cancer makes up for about 7.6% of cancers in females and one of the commonest risk factors is HPV infection, i.e. 83.2% of patients diagnosed with cervical cancer had HPV virus [19] and therefore awareness about HPV virus and vaccinations against infections needs to be promoted. Despite the high number of average hours of sunshine in Malaysia, Malaysians’ awareness about signs and symptoms of skin cancer and risk factors related to sun exposure was low. Different to Australia where melanoma of the skin is a major focus of public health awareness campaigns due it’s high incidence (12% of all cancers (Australian Institute of Health and Welfare, [20])), it seems to be a lesser priority for Malaysia likely due to comparably low incidence rates, i.e. melanoma constitutes 2.7% of cancers in males [21] which may be due to the fact that most Muslims, in particular females, cover their skin and Malaysians generally avoid spending time in the sun. Even though knowledge about smoking as a risk factor was high, about 20% did not recognise it as a risk factor for cancer. This is of concern as smoking has been found to be significantly associated with a number of cancers, in particular, lung, laryngeal, pharyngeal and upper digestive tract cancers [22] and the high proportion of Malaysians who smoke is 35–48.5% in people aged 40 and older [23]. Lung cancer is also the second most common in males (14.4%) and fourth most common in females (6.0%) and nasopharyngeal cancer is the fourth most common cancer in males [21].

Factors significantly associated with both symptom and risk factor recognition in the fully adjusted model were age, ethnicity, education and smoking status. Previous research about colorectal, breast and ovarian cancer awareness also found that Malays recognised more symptoms compared to Chinese or Indian participants, that people who completed secondary education or higher demonstrated greater awareness [7, 8, 24] and that middle-aged participants had higher awareness compared to participants aged 60 years or older [7, 24].

Compared to findings from a study that utilised the ABC tool to assessed cancer awareness in high income countries, which are part of the International Benchmarking Partnership, the findings from this study highlight a significant gap in cancer symptom awareness between Malaysians [25] and participants from high-income countries. In particular, the number of recognised cancer signs and symptoms is much lower in Malaysia (5.5 out of 11 symptoms) compared to 7.71 symptoms recognised by Swedish participants, 8.22 symptoms by UK participants and 8.7 symptoms recognised by Canadian participants [25]. One reason for the marked differences is the increased cancer prevention and early detection activity in high income countries compared to low income countries [26], for example population-based cancer screening is often in place for the most common cancers in high income countries and it is often lacking in low and middle income countries. Furthermore, the population in this study reported much lower experience with cancer (self, friend or family) compared to participants from Western countries (20% and between 80 and 85%, respectively). The low awareness demonstrated here suggests an urgent need for cancer awareness activities to increase in Malaysia as awareness about cancer signs and symptoms is key to early diagnosis and currently cancer in Malaysia is often detected late (Stage 3 or 4) [21], reducing chances of survival.

The major strengths of this study are I) the use of the validated and globally used ABC measurement tool which allows for comparison of cancer awareness with western countries, II) random selection of participants and the moderately large sample size and III) the inclusion of participants from all states across Malaysia aged 40 years or above. A potential limitation of the study is that participants who agreed to complete the survey over the telephone might be more interested in health overall compared to participants who did not agree to participate, which is supported by the low number of current smokers (18.7%) recruited compared to the national average (40%). Furthermore, the sample is not quite representative of the Malaysian ethnic and gender distribution [13] (i.e. males and Chinese are overrepresented). Also, participants attained higher level of education compared to the general public s (i.e. over 75% of the population completed secondary or tertiary education whereas according to the Malaysian census in 2010 about 60% of the population completed secondary or tertiary education [27]). However, as described earlier, compared to other studies, cancer awareness as reported here is still relatively low.

## Conclusion

Findings presented here suggest that there are still significant gaps in knowledge about cancer-related signs and symptoms and risk factors in Malaysia. This research highlights the need for public health interventions in Malaysia to increase cancer symptoms awareness across Malaysia in adults aged 40 years and older who are at higher risk for cancer. In addition, efforts should be made to increase awareness about cancer risk factors (in particular dietary- and lifestyle-related risk factors and HPV vaccination) in Malaysia. Since awareness about smoking as a cancer risk factor and lumps as a cancer warning signs was highest, this seems to suggest that public health interventions are successful in increasing knowledge as ‘stop smoking’ and ‘breast cancer awareness’ campaigns have been a focus of previous public health efforts. Cancers that were previously less promoted need to receive further attention.

## Data Availability

Data is available on request from the corresponding author Prof Tin Tin Su.

## Abbreviations

ABC: Awareness and Beliefs about Cancer (measurement tool)
CATI: Computer-assisted telephone interview
CI: Confidence interval
*n*: Number
SD: Standard deviation
UK: United Kingdom
